# Selenium Metabolism in Cancer Cells: The Combined Application of XAS and XFM Techniques to the Problem of Selenium Speciation in Biological Systems

**DOI:** 10.3390/nu5051734

**Published:** 2013-05-21

**Authors:** Claire M. Weekley, Jade B. Aitken, Lydia Finney, Stefan Vogt, Paul K. Witting, Hugh H. Harris

**Affiliations:** 1 School of Chemistry and Physics, The University of Adelaide, Adelaide, SA 5005, Australia; E-Mail: claire.weekley@adelaide.edu.au; 2 School of Chemistry, The University of Sydney, Sydney, NSW 2006, Australia; E-Mail: j.aitken@chem.usyd.edu.au; 3 X-ray Science Division, Advanced Photon Source, Argonne National Laboratory, Lemont, IL 60439, USA; E-Mails: lfinney@aps.anl.gov (L.F.); vogt@aps.anl.gov (S.V.); 4 Biosciences Division, Advanced Photon Source, Argonne National Laboratory, Lemont, IL 60439, USA; 5 The Discipline of Pathology, Sydney Medical School, The University of Sydney, Sydney, NSW 2006, Australia; E-Mail: pwitting@med.usyd.edu.au

**Keywords:** selenium, cancer, XAS, XFM, selenoproteins, SDS-PAGE

## Abstract

Determining the speciation of selenium *in vivo* is crucial to understanding the biological activity of this essential element, which is a popular dietary supplement due to its anti-cancer properties. Hyphenated techniques that combine separation and detection methods are traditionally and effectively used in selenium speciation analysis, but require extensive sample preparation that may affect speciation. Synchrotron-based X-ray absorption and fluorescence techniques offer an alternative approach to selenium speciation analysis that requires minimal sample preparation. We present a brief summary of some key HPLC-ICP-MS and ESI-MS/MS studies of the speciation of selenium in cells and rat tissues. We review the results of a top-down approach to selenium speciation in human lung cancer cells that aims to link the speciation and distribution of selenium to its biological activity using a combination of X-ray absorption spectroscopy (XAS) and X-ray fluorescence microscopy (XFM). The results of this approach highlight the distinct fates of selenomethionine, methylselenocysteine and selenite in terms of their speciation and distribution within cells: organic selenium metabolites were widely distributed throughout the cells, whereas inorganic selenium metabolites were compartmentalized and associated with copper. New data from the XFM mapping of electrophoretically-separated cell lysates show the distribution of selenium in the proteins of selenomethionine-treated cells. Future applications of this top-down approach are discussed.

## 1. Introduction

Organic and inorganic selenium compounds are dietary sources of the essential element that is critical for the assembly of selenoproteins. Selenium may also reduce the incidence of cancer when taken at supranutritional doses [1]. The average recommended daily intake of selenium is 53 µg per day for women and 60 µg per day for men [1,2]. Evidence from *in vitro* studies, animal experiments and clinical trials suggest that the biological activities of selenium are dependent on the speciation of the metabolites derived from the ingested selenium compound. In order to fully understand the mechanisms of the chemopreventative, anti-cancer and other biological activities of selenium compounds, the relationship between these properties and the chemical form of selenium must be understood. As such, understanding the metabolism and speciation of selenium *in vivo* is an important area of research in selenium biology. The advancement of speciation techniques in the last decade has led to considerable progress in the identification of selenium species in biological systems. In this review we briefly summarize some recent research into *in vivo* selenium metabolism and speciation based on HPLC-ICP-MS and related techniques, before focusing on the application of synchrotron-based X-ray absorption spectroscopy (XAS) and X-ray fluorescence microscopy (XFM) to the same problem.

## 2. The Selenium Speciation Problem

A selenium-replete diet is one that enables the full expression of selenoproteins; proteins that incorporate selenium as selenocysteine (SeCys). In humans, 25 selenoproteins have been identified including the antioxidant glutathione peroxidases (GPx), the thioredoxin reductases (TrxR; which are redox regulators), the iodothyronine deiodinases that are involved in thyroid hormone metabolism and Selenoprotein P (SelP), which transports Se to the brain, among other functions [3,4,5,6,7,8]. The functions of several other selenoproteins are still poorly defined. There is evidence that selenoproteins can both prevent and promote cancer [1,9,10] and it is therefore important to understand the roles of selenoproteins in chemoprevention and carcinogenesis. However, the focus of this review is on the speciation and biological activity of low molecular weight Se metabolites, which have also been implicated in the chemoprevention and anti-cancer mechanisms of Se [2,11,12]. The seminal example of the benefits of Se supplementation is the Nutritional Prevention of Cancer (NPC) Trial. Participants in the trial received dietary supplements in the form of selenised yeast tablets resulting in a reduction in the incidence of prostate cancer and in total cancer incidence [1,3,4,5,6,7,8,13,14,15]. The baseline plasma Se level of almost all of the NPC Trial participants was above that observed to result in maximal expression of plasma selenoproteins [14], which suggests that benefits associated with Se supplementation may be derived from low molecular weight Se metabolites. 

The positive outcomes of the NPC Trial generated impetus for a larger trial. The Selenium and Vitamin E Cancer Prevention Trial (SELECT) provided participants with the same Se dose as in the NPC Trial (200 µg Se/day) in order to test whether it could prevent prostate cancer. Selenomethionine (SeMet) was selected as the form of Se supplementation in the trial over selenised yeast, selenite and monomethylated selenium compounds (e.g., methylselenocysteine or methylseleninic acid) that were also considered [12,16,17]. The trial was discontinued when analysis showed no benefit, nor any prospect of benefit, from Se or vitamin E supplementation alone, or in combination [18,19]. 

Several hypotheses have been proposed to explain the different outcomes of SELECT and the NPC Trial [20,21,22,23]: The choice of SeMet for the supplement is one of them [13,24,25]. SeMet is a major component of selenised yeast, but the selenised yeast tablets used in the NPC Trial contained other selenium compounds in addition to SeMet, such as methylselenocysteine (MeSeCys), that may be more effective in chemoprevention [26,27,28,29]. It is understood that the biological activities associated with Se are dependent on the chemical speciation of Se, but it is not known which selenium compounds are most efficacious in cancer prevention; nor are the anti-cancer and chemopreventative mechanisms of Se fully established. Therefore, identifying the metabolic products of different forms of Se *in vivo* and understanding the relationship between the ingested form of Se, its metabolites and their biological activities is crucial to explaining the anti-cancer effect of Se.

### 2.1. Dietary Selenium Compounds and Their Metabolites

**Figure 1:**
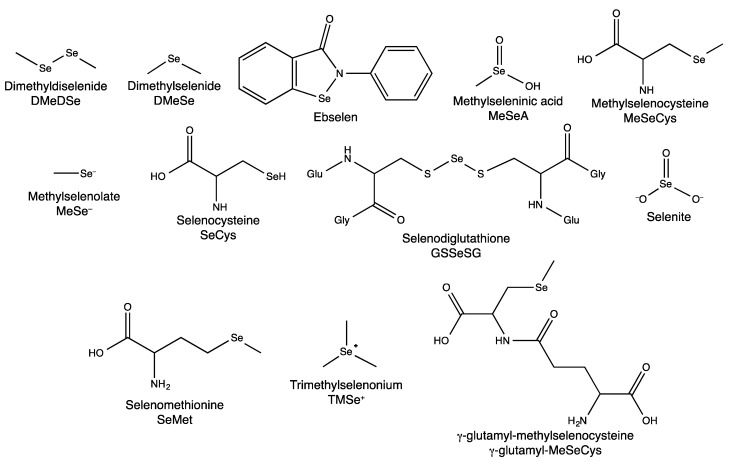
The structures, names and abbreviations of Se compounds referred to in this review.

The current understanding of Se metabolism has been developed over several decades [13,30], with most metabolites initially identified in *in vitro* chemistry studies or in cell-free tissue homogenates through indirect methods. More recently, many metabolites have been identified in mammalian systems by ICP-MS after separation by HPLC, but only a few have been unambiguously identified by molecular mass spectrometry [31,32,33]. The structures of Se compounds pertinent to this review are presented in Figure 1.

**Figure 2:**
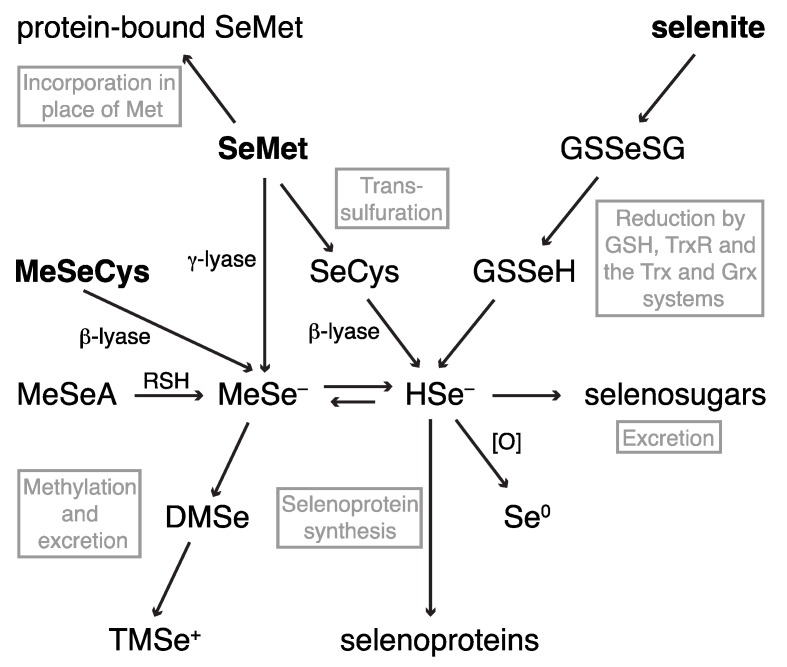
An overview of the metabolism of the dietary selenium compounds selenite, SeMet and MeSeCys. See text for references.

The most commonly studied dietary selenium compounds are selenite, SeMet and MeSeCys. Each of these compounds follows a different metabolic route to a presumed common Se intermediate, from which the selenoprotein synthesis and the Se excretion pathways originate, as shown in Figure 2. The excreted methylated metabolites are the most readily analyzed and dimethylselenide (DMSe), established as the major Se excretory metabolite in breath, was first identified as early as the 1950s [34,35]. Trimethylselenonium (TMSe^+^) was first identified in rat urine in 1969 [36,37] and was considered the major urinary metabolite until the identification of selenosugars in urine by HPLC-ICP-MS and ESI-MS/MS in 2002 [13,38,39,40]. The late Suzuki and co-workers used HPLC-ICP-MS in conjunction with radiolabelling to investigate the efficiency of methylation and demethylation steps in rat tissue homogenates and supernatants incubated with selenium compounds [38,41]. By monitoring the generation of methylated excretion products and selenosugars, they made inferences about the relative efficiency of putative methylselenol (MeSeH; monomethylselenolate, MeSe^−^ at physiological pH [42]) generation by selenium compounds [43,44]. The ability to generate MeSe^−^ may be crucial to the anti-cancer properties of selenium compounds [45,46].

The dietary selenium compounds share some metabolic pathways: the methylation and selenosugar excretory pathways, and the production of selenoproteins. The metabolic routes to the presumed common selenide intermediate, however, differ with Se speciation. As seen for the excreted metabolites, hyphenated techniques that combine separation and detection methods are the techniques of choice for the identification of non-excreted metabolites. However, the identification of low molecular weight selenium compounds in cells and *in vivo* has proved a challenge for speciation chemists.

Inorganic selenite is reduced by glutathione (GSH), TrxR and the thioredoxin and glutaredoxin systems to selenodiglutathione (GSSeSG). GSSeSG is further reduced via a selenopersulfide (GSSeH) to hydrogen selenide (HSe^–^) by the same systems [3,5,6,7,8,47]. GSSeSG has been identified by HPLC-ICP-MS in spiked rat liver cytosols [9,48] and by ESI-MS/MS in cell culture [11,49], but the volatility and reactivity of the intermediate GSSeH and product HSe^–^ make their *in vivo* detection difficult. Speciation analysis of selenite-treated lung cancer cells by Lunøe *et al.* revealed that a significant proportion of Se was bound to protein while the remainder was unchanged [13,50]. The nature of the Se bonding within the protein fraction was not explored. 

The Se analogue of methionine (Met), SeMet, has been shown, in rat liver homogenates, to be metabolised to SeCys via the trans-sulfuration pathway [51,52]. SeCys can then be cleaved by β-lyase to HSe^–^ [16,53]. In addition, SeMet can be adventitiously incorporated into proteins in place of Met [18]. A third pathway is open to SeMet in its cleavage by γ-lyase to MeSe^–^, although the extent to which this pathway operates is uncertain [20,22,48]. In a number of cancer cell lines, Lunøe *et al.* [13,54] found that SeMet largely remained intact and did not react with cytosolic proteins. 

Another selenoamino acid, MeSeCys, has a metabolic pathway distinct from that of SeMet, in that its major metabolite is MeSe^−^, generated by β-lyase cleavage of the amino acid [26,55]. In some cancer cells, MeSeCys was largely unmetabolised, with the generation of dimethyldiselenide (DMeDSe) alone suggesting the generation of MeSe^–^, although MeSeCys was comparatively non-toxic to the cells [13,56]. Tsuji *et al.* [31,57] found that long-term ingestion of MeSeCys by rats led to Se accumulation in the kidney, liver and testes, selenoprotein synthesis and the excretion of TMSe^+^ and selenosugars in urine, in accordance with the prevailing model of Se metabolism [34]. 

Methylseleninic acid (MeSeA) is a simplified form of MeSeCys that is readily reduced to MeSe^–^ in the absence of β-lyase [36]. As such, it is often used in place of MeSeCys in *in vitro* and cell culture experiments. MeSeA has been shown in cancer cells to generate MeSeCys, γ-glutamyl-MeSeCys and DMeDSe, and to interact with proteins [13,38,58]. In addition, the identification of SeMet in MeSeA-treated cells suggests the existence of a currently unknown metabolic pathway that warrants further investigation [38,59].

#### 2.1.1. Hyphenated Techniques and Selenium Speciation

A comparative study of the metabolism of selenite, SeMet and MeSeCys in rat tissues, each labeled with a different stable isotope of Se, is a typical example of the application of hyphenated techniques [60,61]. SeMet and MeSeCys were found to be transported to organs unchanged, but selenite was metabolised before reaching the organs. All of the selenium compounds contributed to SelP production, but selenite was most effective, followed by SeMet and MeSeCys. Finally, based on the differing abilities of each of the selenium compounds to produce TMSe^+^ and selenosugars in the liver and kidneys, the authors were able to argue that the metabolism of MeSeCys was distinct from both SeMet and selenite metabolism; supporting the current model of Se metabolism that suggests MeSeCys is the most important generator of MeSe^–^. Still, some metabolites remained unidentified and the speciation of low molecular weight selenium compounds that lie between the dietary compounds and the excretory compounds was not determined.

The hyphenated techniques have been extensively applied to the analysis of Se speciation in food and selenised yeast [43] and in animal models and human body fluids [45]. However, while offering excellent sensitivity to trace compounds and the ability to separate and unequivocally identify individual compounds, these techniques have the disadvantage of requiring extensive sample preparation (including digestion, extraction and separation steps) of cells and tissues. The digestion and acidification of samples can alter the chemistry of Se, particularly in regards to its oxidation state [47,62]. Furthermore, while the speciation of Se in different tissues can be ascertained, information on the distribution of Se within organs is lost in the homogenization of the samples. An alternative to the hyphenated techniques is required to provide unadulterated *in vivo* Se speciation and distribution information. 

## 3. Synchrotron-Based X-ray Techniques in Elemental Speciation Analysis

Synchrotron-based X-ray techniques are a viable alternative to hyphenated techniques in elemental speciation analysis. The brilliant, penetrating, high-energy X-rays produced by a synchrotron can be tuned to probe a sample for a specific element; taking advantage of the characteristic binding energies of core electrons in the atoms of each element. In X-ray absorption spectroscopy (XAS), a spectrum, similar to a UV/vis spectrum, is generated by scanning across an energy range above and below the absorption edge (the energy at which core electrons are excited) of the element. An X-ray absorption near edge structure (XANES) spectrum is collected within about 100 eV of the absorption edge [48,63,64] as the core electron is excited to a higher energy level. In biological samples, where the concentration of the element of interest is usually low and high sensitivity is required, the fluorescent photons, emitted as higher energy electrons fill the core electron shell, are collected to generate the XANES spectrum. The XANES spectrum reflects the average coordination environment and oxidation state of the element of interest.

At even higher energies above the absorption edge, the core electrons are ejected from the atom and interact with neighboring atoms to generate an oscillating pattern in the spectrum—the extended X-ray absorption fine structure (EXAFS). The coordination number of bound atoms can be estimated (±20%) from the EXAFS spectrum and bond lengths can be determined to within 0.02 Å, although atoms of similar atomic number (e.g., N and O) cannot normally be distinguished [49,65]. A single energy scan of a sample can provide a single spectrum containing both XANES and EXAFS, which can be analyzed independently to determine the speciation of the element of interest. XANES spectra are analyzed by fitting a linear combination of model compound spectra to the sample spectrum, while EXAFS spectra can be analyzed *a priori*. 

The fluorescent photons that are used to create an XAS spectrum are also the basis of X-ray fluorescence microscopy (XFM). Instead of scanning across an energy range (as per XAS), in XFM the energy is held constant above the absorption edge and the position of the sample is scanned to create an elemental map based on the intensity of fluorescence at each position. Crucially, the energy used to excite the element of interest will also excite the elements with atomic numbers lower than that element. Thus a number of elemental maps are generated simultaneously, although the sensitivity to elements at low atomic numbers decreases as the incident energy increases (typically we detect down to sodium). In addition to mapping the distributions of individual elements across cells and tissues, spatial relationships between elements can also be identified and by comparison to elemental standards, the concentrations of the elements can be determined. 

In recent years, XAS and XFM, separately and in combination, have been applied to the study of metals and non-metals heavier than Si in biology. XAS and XFM were used to show that silver was bound to Se in some marine mammals and sulfur in others, probably as a detoxification mechanism [50,66]. Korbas *et al.* [51,64] studied the speciation of methylmercury in human brain tissue from individuals with varying levels of mercury exposure and found, using XAS and XFM, that Se may play a role in mercury detoxification. XAS and XFM are increasingly popular techniques in the field of bioinorganic chemistry—a recent tutorial review offers an overview of the application of XAS to biological samples [53,67] and some examples of XFM in biology are reviewed by Fahrni [68,69], while the application of both XAS and XFM to the study of metal-containing drugs has been reviewed by Aitken *et al.* [48]. 

### The Use of Synchrotron-Based X-Rays to Investigate Selenium in Biology

amenable to XAS and XFM techniques. XANES spectra of organic Se compounds are distinct from each other and from inorganic selenium compounds, which allows for linear combination fitting of mixtures of selenium compounds found in biological samples [54]. However, in comparison to hyphenated techniques, XAS is disadvantaged by its lack of sensitivity at low concentrations and its inability to detect trace components. It is limited to determining the coordination and oxidation environment of Se, rather than the exact identity of the selenium compound. Care must also be taken to prevent photo-reduction of selenium compounds in high oxidation states [55]. The advantages of XAS include the ability to collect two independent sources of information from XANES and EXAFS spectra and the ability to apply the same technique to a variety of samples with very minimal sample preparation. All selenium compounds, whether protein-bound, hydrophobic or ionized, can be interrogated without the need for multiple extraction and separation procedures, using a single sample preparation method. 

The simultaneous mapping of biologically relevant elements in intact biological samples, allowing for the observation of relationships between different elements, is a strength of XFM. The atomic number of Se (*Z* = 34) and its corresponding 1s ionization energy make it ideal for elemental mapping as the endogenous elements P, S, Cl, K and Ca are all mapped simultaneously. Importantly, the transition metals Fe, Cu and Zn, with energies of their core electron shells not much lower than Se are also mapped with good sensitivity. In combination, XAS and XFM provide complimentary information on elemental speciation and distribution that can provide novel information, drive new research directions and can be applied to *in vitro* systems, biological fluids, cells and tissues alike.

Several investigators have applied either XAS or XFM to the study of Se in biology. Wang *et al.* [56] showed that Cu(I) binds to selenoamino acids in phosphate buffer, using both Se K-edge and Cu K-edge XAS to characterize the Se-Cu bonds in addition to NMR and DFT studies. A natural extension of this study would be to attempt to identify and characterize this interaction in cells: a study to which XAS is well-suited. The speciation of Se in Se-enriched yeast was investigated using both XAS and HPLC-ICP-MS where the results from each technique were in agreement, although the restricted model compound library and absence of an EXAFS spectrum limited the information that could otherwise be derived from XAS [57]. XANES has been applied to the study of Se metabolism in rainbow trout hepatocytes exposed to selenite, selenate or SeMet [70] and extended to the determination of Se speciation in tissues from rainbow trout exposed to SeMet [71]. 

The localization of Se in a neuronal cell model, treated with the Se drug ebselen, was observed using XFM [58]. The technique revealed the effects of ebselen on Mn, Fe, Cu and Zn homeostasis in addition to Se distribution. Gladyshev and coworkers have used XFM of tissues from knock-out and wild-type animals combined with traditional immunohistochemical techniques to investigate the roles of selenoproteins. In addition to mapping the distribution of Se in spermatids, the roles of mitochondrial GPx4 and SelP in mouse spermatogenesis were unraveled using XFM in coordination with traditional biochemical techniques [59]. Similarly, the distributions of Se in mouse liver and the kidneys of mice and naked mole rats were mapped using XFM to reveal relationships between tissue levels of Se and the expression of GPx1, GPx3 and SelP [60]. In a rat model of myoglobinuria, XFM was used to show a relationship between Se distribution and GPx1 in the kidneys of rats fed a high-Se diet as selenite [72]. XAS and XFM, in combination with other speciation, chemical and biochemical techniques can clearly be applied to a wide variety of systems and problems involving Se.

## 4. A Top-Down Approach to Investigating Selenium in Biology

Herein, we present a top-down approach to the investigation of Se in biology: studying the speciation of metabolites of supranutritional doses of selenium compounds in human cancer cells, using XAS and XFM techniques that, in combination, provide insight into the speciation and distribution of Se in bulk and single cell samples. The nature of each X-ray technique dictates the type of sample that can be interrogated. The X-rays available at X-ray absorption beamlines are typically delivered in beams on the order of millimeters in width to provide sufficient photon flux for the collection of XANES and EXAFS spectra. Thus, bulk cell samples, containing approximately 10^5^–10^6^ cells [73], are required for XAS measurements. The XANES and EXAFS spectra from those bulk samples are averages of all of the regions within all of the cells. In contrast, XFM beamlines are designed to produce beams from a few hundred nanometers to a few micrometers in diameter allowing the elemental mapping of single cells at sub-micron resolution. XAS may also be collected from samples at some XFM beamlines, thereby providing localised chemical speciation information, although the low concentrations and lower flux (relative to the larger, traditional XAS beams) result in low resolution spectra. As such, microprobe-XAS (µ-XAS) techniques are limited to regions with high concentrations of the element of interest. The combination of bulk XAS, µ-XAS and XFM techniques is capable of providing bulk and localised speciation information as well as elemental distributions in single cells.

### 4.1. Selenium Speciation Dictates Distribution in Human Lung Cancer Cells—Findings from XFM

**Figure 3:**
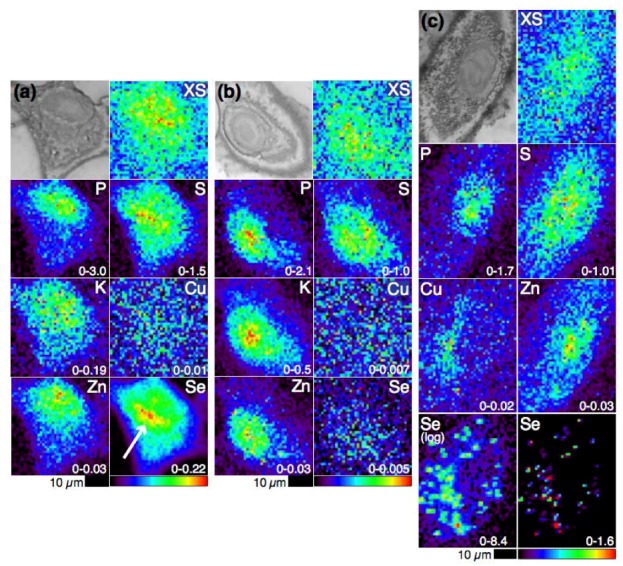
Optical micrographs (top left), scattered X-ray (XS) and XFM elemental distribution maps of A549 cells treated with (**a**) 50 µM SeMet (the white arrow indicates the perinuclear accumulation of Se), (**b**) 50 µM MeSeCys or (**c**) 5 µM selenite. The range of elemental area densities (quantified from standards and expressed in micrograms per square centimeter) is given in the bottom corner of each map.

Human cancer cell lines, which are more sensitive to Se than normal cells [62], are relevant models for the study of the biological activities of Se metabolites. As they are more complex than *in vitro* systems, but less complex than animal models, they are appropriate model systems for the first application of our top-down approach. XFM revealed that Se distribution in A549 human lung cancer cells is dependent upon the speciation and concentration of the selenium compounds with which they are incubated (Figure 3), which supports the model of distinct metabolic pathways of dietary selenium compounds [63,64,74]. It was observed that SeMet-treated cells readily accumulate Se: after 24 h a 70-fold increase in Se versus vehicle alone control cells was observed in 10 µM SeMet-treated cells and a 270-fold increase was observed in 50 µM SeMet-treated cells. Fifty micromolar MeSeCys-treated cells, despite the much higher toxicity of MeSeCys to the cells than SeMet, contained only four-fold more Se than control cells. In the resulting Se elemental maps the cells were virtually indistinguishable from the background (similar to what was observed in maps of control cells). In stark contrast, elemental maps of SeMet-treated cells showed that Se was present throughout the cells with a strong accumulation in the perinuclear region (indicated in Figure 3a). The perinuclear accumulation was a feature shared with sulfur that, given the ability of SeMet to be adventitiously incorporated into proteins in place of methionine, suggests that this perinuclear Se accumulation may be in the endoplasmic reticulum, a site of protein synthesis.

The difference in the Se contents of the SeMet- *versus* the MeSeCys-treated cells may be explained by a difference in speciation and metabolism. Volatile metabolites produced by MeSeCys-treated cells may have escaped (a hypothesis that fits with the observation of volatile DMeDSe forming in the head space of hepatocytes incubated in MeSeA [65]) or, during sample preparation, Se species present in MeSeCys-treated cells may have been washed out, but the Se species present in SeMet-treated cells were not. The latter explanation points to the importance of sample preparation in XFM. Although sample preparation is minimal compared to the preparation required for HPLC-ICP-MS, it may still interfere with the sample by selectively mobilizing and redistributing or washing out metals and this possibility must be considered when analyzing XFM data, as emphasized by the chemical alterations that have been observed in formalin-fixed versus cryofixed brain tissues [66].

The most distinctive Se distribution was observed in selenite-treated cells. Selenite is the most toxic of the three selenium compounds discussed here. The IC_50_ of selenite at 72 h (5 µM) in these human lung cancer cells is 20- to 100-fold lower than the IC_50_s of MeSeCys and SeMet, respectively [64]. Twenty-four hours selenite treatment at 1 µM resulted in a Se concentration that was almost indistinguishable from background levels. However, a 5 µM selenite treatment resulted in a 250-fold increase in Se, similar to that observed in 50 µM SeMet-treated cells, but with a very different distribution. Selenium was found to have accumulated in many small regions (a few microns in diameter) in the cytosol. In addition, Cu was found to share this punctate distribution and undergo a two-fold increase in concentration, which suggested that Cu is either complicit in the cytotoxic mechanism of selenite or is part of a cellular defense mechanism against selenite-induced toxicity. Recent work suggests that the increased Cu concentration is due to the upregulation of SOD1 in response to increased ROS generation that occurs 24 h after selenite treatment [75].

Copper sulfate has previously been found to prevent Se toxicity when provided to cells [67] and rats [68] at the same time as selenite, but this was the first observation of a Cu and Se association in cells treated with selenite alone. The observation of the Se and Cu accumulation in selenite-treated cells patently demonstrates the usefulness of XFM in generating multiple elemental maps: the destruction of samples inherent in the use of hyphenated techniques means that these types of elemental associations, where the associated elements are not necessarily bound within the same compound, cannot be identified by these techniques. 

### 4.2. Selenium Speciation in Bulk Cell Pellets and Single Cancer Cells

**Figure 4:**
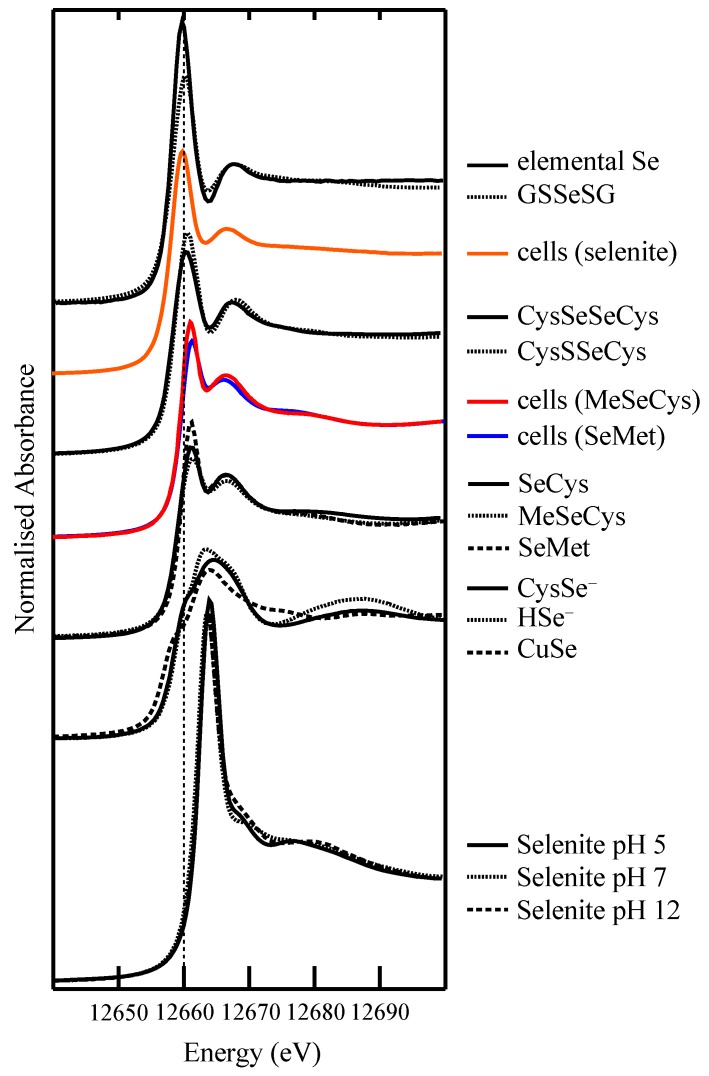
Library of Se K-edge X-ray absorption spectra of model selenium compounds used in the linear combination fitting of experimental spectra. XANES spectra of cells treated with 100 µM SeMet (*blue*), 100 µM MeSeCys (*red*) or 5 µM selenite (*orange*) are also shown for comparison.

Speciation information from XAS augments the distribution information from XFM and helps to explain why Se distribution differs between cells treated with different dietary selenium compounds [63,64,74]. XANES and EXAFS spectra both show that SeMet- and MeSeCys-treated cells have speciation profiles that are completely distinct from the speciation profile of selenite-treated cells. Fits to EXAFS spectra are generated *a priori*, but fits to XANES spectra require a model compound library. Our model compound library, shown in Figure 4, covers the most likely Se coordination environments in biology, which are clearly distinguishable based on the coordination environment and oxidation state: Se–Se–Se, S–Se–S, R–Se–R, R–Se–Se–R, R–Se^–^, O–Se–O, R–Se–S–R. The models used to fit the experimental XANES spectra are drawn from the library after principal component analysis and target transformation indicate the number and type of model compounds to use in the multiple linear regression analysis. As mentioned earlier, XANES indicates the coordination environment of the Se atom, but does not identify specific Se species. Comparison of SeMet, MeSeCys and SeCys XANES spectra, for instance, attests to the similarity of the spectra produced by different selenium compounds with the same coordination environment around the Se atom. Thus, the three amino acids are collectively considered R–Se–R compounds, as it is unlikely that a mixture of these compounds can be reliably deconvoluted by linear combination fitting. 

SeMet-treated A549 bulk cell pellets were shown by XANES and EXAFS analysis to contain carbon-bound Se species as did the perinuclear region of the cells when probed by µ-XANES (while noisy, direct visual comparison of these spectra to model compounds clearly showed they shared their key features with R–Se–R models) [63]. Taken together, these data suggest that most of the Se in these cells is found protein-bound as SeMet or SeCys. XANES fitting revealed that R–Se–R species constituted 80%–85% of the Se in MeSeCys-treated cells, with the remaining 15%–20% composed of a diselenide species. The concentration of Se in the cells was too low to confirm with EXAFS, highlighting the low sensitivity of this technique and so the experiment was repeated in SH-SY5Y human neuroblastoma cells, in which Se was more highly concentrated and to which MeSeCys was less toxic [74]. The XANES results reflected those found in the corresponding A549 cells—85%–90% R–Se–R and 10%–15% R–Se–Se–R—and were supported by the results of EXAFS analysis, which clearly revealed a Se scatterer with a Se–Se bond length of 2.36 Å in addition to carbon scatterers at a Se–C bond length of 1.96 Å. By comparison, a Se–C bond was the only contribution to the EXAFS spectrum of SeMet-treated SH-SY5Y cells. 

The presence of diselenide species in the MeSeCys-treated cells, but not SeMet-treated cells, suggests that MeSeCys generates metabolites that alter the redox balance of the cell to a more oxidising environment, but SeMet does not. The implication, based on the accepted Se metabolism pathway is that MeSeCys is a better generator of the putative reactive oxygen species (ROS) generator, MeSe^–^ [76]. Methylselenol (R–Se–H/R–Se^–^) is not present in our model compound library, but would be expected to have a similar spectrum to SeCys, which is included in the library in its protonated (R–Se–H) and deprotonated form (R–Se^–^); the latter is predominant at physiological pH [77]. A diselenide, DMeDSe, has been identified by membrane inlet mass spectrometry as a volatile metabolite of MeSeA-treated rat hepatocytes [65] and by gas chromatography and mass spectrometry in MeSeA-treated human lymphoma cells [38], but diselenides were not identified *within* the cells. The presence of diselenide within the A549 cells may indicate the presence of DMeDSe and/or the oxidation of selenols within the cells, the latter possibility having implications for protein function.

The predominantly R–Se–R speciation of Se in the selenoamino acid-treated cells is in contrast to the mostly inorganic species observed in cells treated with 5 µM selenite in A549 cells [64]. After 1 h, all of the selenite had been metabolised, contrary to what Lunøe *et al.* [13] found in prostate, colon and leukemia cancer cells, but in accordance with the *in vitro* chemistry, the abundance of GSH in cells and findings from an animal model [61]. The presence of S–Se–S and elemental Se species after 24 h conforms to the reductive metabolism pathway observed *in vitro*. Previously, elemental Se had not been identified in mammalian cells, which may be due to its insolubility—a large insoluble Se fraction of selenite-treated rat hepatocytes was observed by Gabel-Jensen *et al.* [78], which LC-ICP-MS is incapable of analyzing. HSe^–^ was not identified in the XANES spectra of these, or the SeMet- or MeSeCys-treated cells, which suggests that, at best, it is a very minor component (<5%) of the cells, in keeping with its reactivity and volatility.

In addition to the S–Se–S and elemental Se species, which were identified in both XANES and EXAFS spectra of selenite-treated cells, R–Se–R and diselenide species were identified in XANES spectra (oscillations in the EXAFS due to carbon scatterers are expected to be overwhelmed by the heavy and abundant S and Se scatterers in the sample). Notably, the proportion of the organic and diselenide species changed significantly between 24 and 48 h when the proportion of R–Se–R decreased and the proportion of R–Se–Se–R increased, indicating a change to a more oxidising environment that coincided with a substantial decrease in cell viability. The single cells imaged by XFM were on the precipice of this change, which points to the importance of the distributions of Se and Cu in the cells. From the regions of Se accumulation, µ-XANES spectra were collected and comparison to model compound spectra revealed that these regions were composed predominantly of elemental Se (and perhaps S–Se–S species), which, unless nano-sized, is considered biologically inert. The Se concentration outside of these regions was too low to collect XANES spectra, but it can be inferred from the bulk XANES results that the organic species and diselenides dominate in the remainder of the cell. 

Selenite is known to induce the generation of the superoxide anion radical and other ROS in cells [79,80]. Our recent work (unpublished data) indicates this is the case in 5 µM selenite-treated A549 cells and suggests that SOD1 is upregulated in response to selenite insult, supporting the hypothesis that the response of Cu to selenite insult is part of a protective mechanism against oxidative damage. The possibility of Cu directly bonding to Se in the cells was considered and tested by attempting to fit Cu and Se scatterers to Se EXAFS and Cu EXAFS of the cells, respectively. There was no evidence of Cu bonding in the Se EXAFS and the presence of Se in the Cu EXAFS was ambiguous [75]. Moreover, despite its presence in the model compound library, CuSe was not a component of the Se XANES spectrum. Taken together with the fact that the increase in Cu was much less than the increase in Se, we are confident that the colocalisation of Se and Cu in the selenite-treated cells is not due to any significant intracellular Cu–Se bonding.

Though limited in its sensitivity and ability to fully identify Se metabolites, XAS in combination with XFM has enabled us to identify links between speciation, distribution and biological activity that could not be identified using the traditional hyphenated techniques.

### 4.3. Extending XFM to Selenium in Electrophoretically-Separated Cell Lysates

Imaging of single cells with XFM reveals the spatial distribution of elements, but by combining electrophoresis with XFM, the distribution of elements amongst proteins can be observed. Finney *et al.* [81] recently demonstrated the application of XFM to electrophoretically-separated proteins using two examples: investigating the binding of Cr to blood serum proteins *in vitro*, with the application of µ-XANES to determine Cr speciation; and examining the effects of oxygen depletion on the speciation of Fe in a bacterium. The combination of these two techniques has the potential to help identify new metalloproteins [82] and investigate the effects of changing conditions on the binding of metals by proteins. 

The challenges in conducting XFM of electrophoretically-separated metalloproteins have been well-described by Finney *et al.* [81], particularly with respect to the difficulty in maintaining non-covalent bonds between metals and proteins while achieving good separation using native-PAGE. The benefits of working with Se in this context is that we expect that the majority of, if not all, Se associated with proteins to be bound covalently and so the traditional SDS-PAGE technique, in which detergents, reductants and chelators are used to denature proteins, can be used without trepidation. Nonetheless, it would be of interest to determine if other metals are associated with Se-containing proteins and so minimally disruptive SDS-PAGE (avoiding heating, reductants and chelators, but using some detergent) and native PAGE may still prove useful. Here, we present the first example of XFM imaging of Se in electrophoretically-separated cell lysates.

For a full description of the experimental methods see Supplementary Information. Briefly, A549 cells were treated with SeMet, MeSeCys or selenite for 24 h, lysed with either SDS-containing or native lysis buffer, applied to 4%–20% gradient Bio-Rad mini-PROTEAN TGX gels and separated electrophoretically before blotting onto a PVDF membrane using a wet transfer system. All solutions were made using Milli-Q water and Teflon tweezers were used to prevent metal contamination. The gels were prepared in duplicate with one stained with Ponceau S to visualize proteins, while the other remained unstained for XFM. Imaging was performed at beamline 8-BM-B at the Advanced Photon Source, Lemont, IL, USA. The X-ray beam was tuned to 12.8 keV and passed through a pinhole. Full X-ray spectra were collected by a four-element silicon drift detector (Vortex, SII Nanotechnology, Northridge, CA, USA) every 0.5 mm step over a 2 s dwell time. Spectra were fitted and images were processed using MAPS software [83].

**Figure 5:**
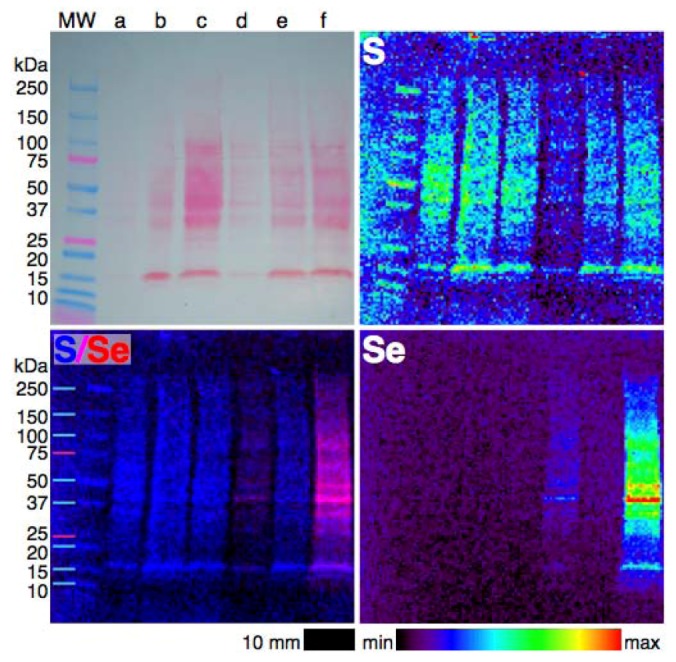
Photograph (top right) and XFM elemental distribution maps of S, Se and an overlay of S and Se in an SDS-PAGE blot of the lysates of cells treated with selenium compounds for 24 h. The wells contain lysates of cells treated with (**a**) 5 µM selenite, (**b**) PBS alone (as vehicle control), (**c**) 50 µM MeSeCys, (**d**) 50 µM SeMet, (**e**) 1 µM selenite or (**f**) 100 µM SeMet. The color scheme indicates the relative concentration within each elemental map.

Selenium was detected in the lysates of SeMet-treated cells separated by SDS-PAGE (Figure 5), minimally disruptive SDS-PAGE (Figure S1) and native-PAGE (Figure S2), but Se was not detectable in either MeSeCys- or selenite-treated cell lysates, despite some of those wells containing similar or greater protein levels (as judged by visual comparison of the intensity of the staining and sulfur maps of each well) than the SeMet-treated cell lysates. No metals were detected within the protein bands on any of the blots, which may indicate that the native-PAGE conditions used were not adequate for retaining metal-protein bonds or that the levels of metalloproteins were not detectable. The Se present in the SeMet-treated cell lysates must be covalently bound in either SeMet or SeCys residues, as expected from the metabolic pathway of SeMet, the results of XAS speciation analysis of whole cells and the conditions of SDS-PAGE. The ubiquitous presence of Se in proteins from 15 to 250 kDa in size suggests that SeMet has been adventitiously incorporated into proteins that, under normal circumstances, contain Met, in keeping with the hypothesis drawn from the observation of an association between Se and S in intact SeMet-treated cells. The inability to detect Se in the other cell lysates has three possible explanations: (1) MeSeCys and selenite are predominantly metabolised into low molecular weight selenium compounds, such as the elemental Se identified in selenite-treated cells; (2) selenoproteins, even in cells with high levels of Se, are below the detection limit of the technique and the high levels of Se in SeMet-treated cell lysates are due only to the adventitious incorporation of SeMet into proteins; or (3) Se is non-covalently bound to MeSeCys- and selenite-treated cell lysates and was cleaved even during native-PAGE.

The qualitative experiment described above is a proof of concept that, at least in SeMet-treated cells, Se can be detected by XFM in cell lysates. By increasing the protein concentration and experimenting with treatment concentrations and optimizing electrophoresis conditions and with the continued improvement of beamlines adapted to this experiment, it may be possible to detect selenoproteins in the lysates of MeSeCys- and selenite-treated cells. The combined electrophoresis and XFM technique is already being expanded into 2D gel electrophoresis [82], and thus has the potential to be applied to the discovery of metals associated with selenoproteins or Se associated with proteins.

## 5. Conclusions

A simplistic view of the Se metabolic pathway would seem to suggest that all Se compounds eventually produce the same metabolites and could be considered equal, but we know that dietary selenium compounds have disparate abilities to generate different Se metabolites—both those considered key metabolites, such as MeSe^−^, and metabolites currently held to be biologically inert, such as elemental Se. Thus, the ability to identify and measure metabolites of dietary selenium compounds *in vivo* is crucial to our understanding of the biological activity of these potentially useful compounds. The results of our XAS and XFM experiments clearly demonstrate the distinct cellular fates of SeMet, MeSeCys and selenite.

In comparison to the traditional hyphenated techniques used in Se speciation analysis, XAS lacks sensitivity and the ability to fully characterize Se species. However, the combination of XAS and XFM techniques is able to provide unique insights into the relationship between Se speciation and distribution and biological activity, with the important advantage of requiring minimal sample preparation.

The findings of our combined XAS and XFM studies summarised in Table 1 show that some key properties of dietary selenium compounds, previously studied *in vitro*, are upheld in intact cells subjected to minimal sample preparation, these properties are: (1) the speciation and distributions of the investigated dietary selenium compounds within cells are distinct from each other, (2) metabolites of MeSeCys and selenite significantly modify the redox status of cells, whereas SeMet does not and (3) selenite follows the reductive metabolism pathway to elemental selenium. More generally, our results show that organic Se metabolites (R–Se–R species) are readily accumulated and distributed widely through the cell, probably as a result of their adventitious or specific incorporation into proteins, whereas the inorganic Se metabolites (elemental Se and possibly S–Se–S) are compartmentalized, which may be due to their toxicity or lack of a specific cellular export mechanism.

**Table 1:** A summary of the speciation and distribution of Se in human lung cancer cells treated with dietary Se compounds, as determined by XAS and XRF techniques [63,64,74].

Se supplement	Speciation of Se metabolites	Distribution of Se metabolites
SeMet	R–Se–R	throughout the cells, but particularly concentrated in the perinuclear region bound to proteins
MeSeCys	mostly R–Se–R significant R–Se–Se–R component	uncertain
Selenite	elemental Se, S–Se–S and R–Se–Se–R some R–Se–R	elemental Se concentrated in small regions throughout the cytosol Se associated with, but not bound to, Cu

### Future Directions

It is clear that these complimentary XAS and XFM techniques can be usefully applied to the problem of Se speciation, leading us to ask new questions and reiterating the need to address extant questions in Se metabolism: is the S and Se colocalisation in the perinuclear region due to adventitious incorporation of SeMet, and if so what are the consequences for these proteins and the cell? (A recent study of SeMet incorporation into amyloid sequences shows that it affects the self-assembly and toxicity of the sequences, suggesting that the level of SeMet incorporation into proteins could have consequences, positive and negative, *in vivo* [84].) What volatile or mobile metabolites have been lost from the MeSeCys-treated cells and are these important in understanding the biological activity of MeSeCys? Is the increase in and colocalisation of Cu with Se part of a defensive response to the insult of selenite metabolites? Where is elemental Se being compartmentalized and does it play any role in the toxicity of selenite towards cancer cells? 

Questions will multiply as this work is extended *in vivo*: while determining speciation in intact cells is an improvement on cell-free models, selenium compounds can be metabolised in bodily fluids and their metabolism varies between organs [61]. The obvious extension of this work is into animals and models of disease, and while these more complex systems will pose fresh challenges, the sample preparation will remain minimal and the collection and analysis of the spectra and images will remain essentially the same. We have begun to extend our work into this area with the XFM investigation of Se distribution in the kidneys of rats subjected to a model of myoglobinuria [72]. 

XAS and XFM techniques continue to advance with improvements to hardware and software, and further development of the techniques. Faster detectors allow the mapping of larger areas at higher resolution with smaller beams on the order of nanometers to probe subcellular structures. Developments in analysis software and techniques improve and expand the information that can be gleaned from XFM data. The mapping of chemical speciation, the ultimate integration of XAS and XFM techniques, has already been demonstrated in the mapping of organic and inorganic Se species in plants [85] and also in the mapping of several different S species in plants [86] and is clearly suited to the mapping of Se species in mammalian systems. Hardware and software improvements are making speciation mapping more accessible as well as allowing the mapping of larger areas of samples so that hundreds of cells, as opposed to a handful, from one sample can be imaged. 

The top-down approach described here is not capable of solving the entire puzzle of Se metabolism and the relationship between Se speciation and biological activity. However, the combination of XAS and XFM techniques is complimentary to traditional biochemical investigations and hyphenated techniques. The results we have presented here both conform to the current understanding of Se metabolism and biological activity, while revealing Se species and Se distributions that pose more questions and provide new leads for research into the complex relationship between Se speciation and biological activity. 

## Supplementary Files

Supplementary File 1 Supplementary Information (PDF, 126 KB)
