# Supplementary material for: Selenium Metabolism in Cancer Cells: The Combined Application of XAS and XFM Techniques to the Problem of Selenium Speciation in Biological Systems
Source: Nutrients. 2013 May 21;5(5):1734–56. doi: 10.3390/nu5051734 (PMC3708347; doi:10.3390/nu5051734)
Supplement: Supplementary File 1 — Supplementary Information (PDF, 126 KB) [file nutrients-05-01734-s001.pdf]

Supplementary Information

S1. Experimental

S1.1. Preparation and Electrophoresis of Cell Lysates

A549 human lung cancer cells were grown and treated with selenocompounds for 24 h at the concentrations indicated in Figure 1 and as described in Weekley *et al.* [1]. At the end of the treatment, cells were rinsed with ice-cold PBS (3 × 5 mL) and lysed in ice-cold lysis buffer (~1 mL/10^7 cells). All solutions described herein were prepared using MilliQ water and Teflon tweezers were used to manipulate materials to minimise metal contamination.

Samples intended for separation by SDS-PAGE were lysed at 4 °C in a SDS lysis buffer (2% w/v SDS; 1 mM sodium orthovanadate; 10 mM EDTA; 20 mM Tris; 0.9% NaCl, pH 7.4; 1 mM protease inhibitor cocktail (P8340, Sigma Aldrich, Castle Hill, Australia)), added fresh with constant agitation for 30 min and the supernatant was collected by microcentrifugation. Samples intended for native-PAGE and minimally-disruptive SDS-PAGE were lysed in a native lysis buffer (62.5 mM Tris, pH 6.8; 1 mM PMSF, added fresh) by three freeze-thaw cycles in liquid nitrogen and hot water and the supernatant was collected by microcentrifugation at 4 °C. Protein concentrations were determined by the bichinchoninic acid assay using a commercial kit (Sigma, Castle Hill, Australia). Samples were stored at 203 K.

Cell lysates were diluted in Tris-HCl loading buffer (final buffer concentrations approximately 31 mM Tris, pH 6.8; 12.5% glycerol; 0.05% bromophenol blue; 1% SDS; 100 mM DDT, added fresh) and applied, next to a lane of molecular weight marker (Precision Plus Protein Dual Xtra protein standard, Bio-Rad), to two 4%–20% gradient Bio-Rad mini-PROTEAN TGX gels prepared in parallel such that approximately 20–30 μg of protein was loaded in each well. SDS-PAGE samples were heated at 100 °C for 3 min before loading and were subjected to electrophoresis at 120 V for 1 h in Tris-glycine running buffer (25 mM Tris; 192 mM glycine; 0.1% SDS, pH 8.3). Gels were blotted onto a PVDF membrane (Immun-Blot, Bio-Rad) using a wet transfer system with Tris-glycine running buffer (25 mM Tris, 192 mM glycine, 20% methanol) at 100 V for 1 h.

Minimally disruptive SDS-PAGE and native-PAGE samples were applied to gels as described for SDS-PAGE samples, but without prior heating and in native loading buffer (lacking the SDS and DTT of the SDS loading buffer described above). Minimally-disruptive SDS-PAGE samples were subjected to electrophoresis at 100 V for 1 h in Tris-glycine running buffer (25 mM Tris; 192 mM glycine; 0.1% SDS, pH 8.3). Native PAGE samples were subjected to electrophoresis at 100 V for 80 min in native Tris-glycine running buffer (25 mM Tris; 192 mM glycine, pH 8.3). Minimally disruptive SDS-PAGE and native-PAGE gels were blotted onto a PVDF membrane as described for SDS-PAGE, but methanol was not present in the blotting buffer.

One membrane from each pair of gels run in parallel was stained with Ponceau S solution while the other was left unstained for XFM.

S1.2. X-ray Fluorescence Microscopy

Imaging was performed at beamline 8-BM-B at the Advanced Photon Source, Lemont, IL, USA. The X-ray beam was tuned to 12.8 keV and passed through a pinhole (spot size on sample, 0.5 mm).

Full X-ray spectra were collected by a four-element silicon drift detector (Vortex, SII Nanotechnology) every 0.5 mm step over a 2 s dwell time. Spectra were fitted and images were processed using MAPS software [2].

Figure S1. Photograph (top right) and XFM elemental distribution maps of S, Se and an overlay of S and Se in a minimally disruptive SDS-PAGE blot of the lysates of cells treated with selenocompounds for 24 h. The wells contain lysates of cells treated with (**a**) 5 µM selenite, (**b**) PBS alone (control), (**c**) 50 µM MeSeCys, (**d**) 50 µM SeMet, (**e**) 1 µM selenite or (**f**) 100 µM SeMet. The colour scheme indicates the relative concentration within each elemental map.

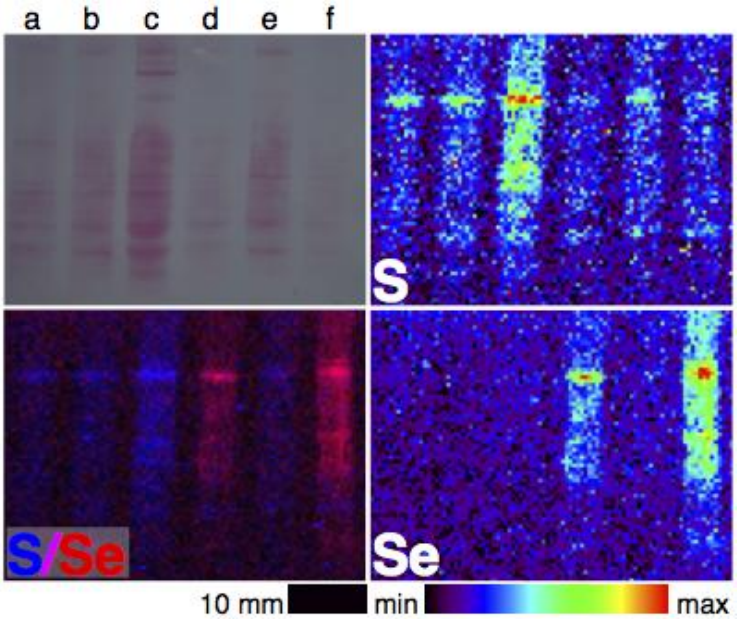

Figure S2. Photograph (top right) and XFM elemental distribution maps of S, Se and an overlay of S and Se in a native-PAGE blot of the lysates of cells treated with selenocompounds for 24 h. The wells contain lysates of cells treated with (**a**) 5 μM selenite, (**b**) PBS alone (control), (**c**) 50 μM MeSeCys, (**d**) 50 μM SeMet, (**e**) 1 μM selenite or (**f**) 100 μM SeMet. The colour scheme indicates the relative concentration within each elemental map.

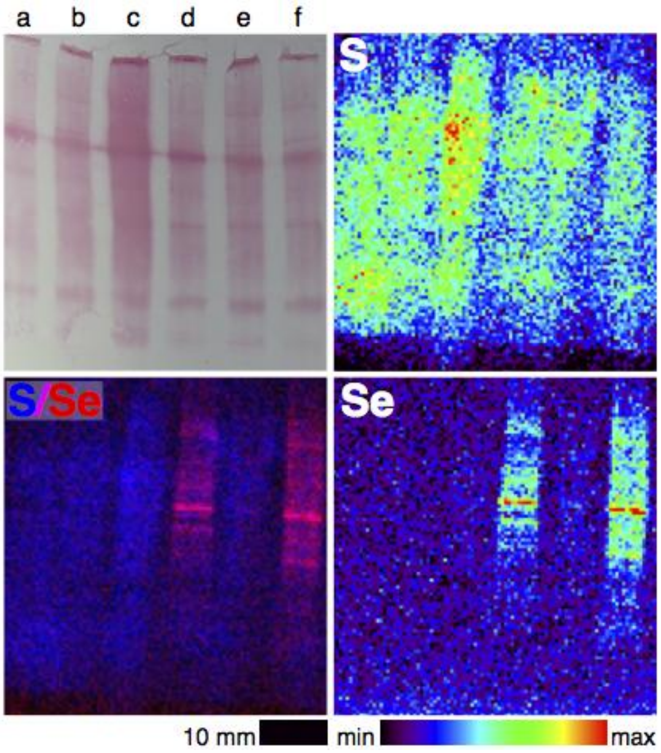

References

1. Weekley, C.M.; Aitken, J.B.; Vogt, S.; Finney, L.A.; Paterson, D.J.; de Jonge, M.D.; Howard, D.L.; Musgrave, I.F.; Harris, H.H. Uptake, distribution, and speciation of selenoamino acids by human cancer cells: X-ray absorption and fluorescence methods. *Biochemistry* **2011**, *50*, 1641–1650.
2. Vogt, S. MAPS: A set of software tools for analysis and visualization of 3D X-ray fluorescence data sets. *J. Phys. IV* **2003**, *104*, 635–638.

© 2013 by the authors; licensee MDPI, Basel, Switzerland. This article is an open access article distributed under the terms and conditions of the Creative Commons Attribution license (http://creativecommons.org/licenses/by/3.0/).
